# Parliamentary amendments aimed at the Brazilian Unified National Health System and the reelection of municipal mayors in Brazil in 2024

**DOI:** 10.1590/0102-311XEN220924

**Published:** 2025-05-19

**Authors:** João Gabriel Ribeiro Pessanha Leal, Luciana Dias de Lima, Frederico Bertholini Santos Rodrigues, André Schimidt da Silva

**Affiliations:** 1 Escola Nacional de Saúde Pública Sergio Arouca, Fundação Oswaldo Cruz, Rio de Janeiro, Brasil.; 2 Instituto de Ciência Política, Universidade de Brasília, Brasília, Brasil.

**Keywords:** Health System Financing, Unified Health System, Legislative, Politics, Municipalities, Financiación de los Sistemas de Salud, Sistema Único de Salud, Poder Legislativo, Política, Municipios

## Abstract

In Brazil, as of 2016, there has been an increase in the share of parliamentary amendments (PAs) in the federal budget, transferred from the Brazilian Ministry of Health to municipalities to fund the Brazilian Unified National Health System (SUS). This article analyzes the association between PAs focused on SUS and the reelection of municipal mayors in 2024. The research used public secondary data, involving 2,818 municipalities. The reelection of mayors was related to the per capita average of amendments transferred from the Brazilian National Health Fund, from January 2021 to October 2024. Hierarchical logistic regression was used, controlling individual and political factors of candidates, and socioeconomic and demographic municipal factors. The results indicate a positive and statistically significant association between the increase in resources for health amendments and the probability of reelection of mayors in 2024 in all models and cutoffs. Note that the values of amendments were considerably higher in municipalities with up to 20,000 inhabitants compared to the other municipalities. Possibly, the amendments consolidate the image of city mayors as capable of attracting resources, strengthening their electoral support in a context of quota of federal expenses. The political use of the amendments contrasts with the criteria adopted for the transfer of resources from SUS programs and may weaken the coordination of the Brazilian Ministry of Health.

## Resumen

En Brasil, a partir de 2016, hubo un aumento en la participación de las enmiendas parlamentarias (EPs) en el presupuesto de la Unión transferidas del Ministerio de Salud a los municipios para financiar el Sistema Único de Salud (SUS). En este artículo se analiza la asociación entre las EP destinadas al SUS y la reelección de alcaldes municipales en 2024. La investigación utilizó datos secundarios públicos, que involucraron a 2.818 municipios. La reelección de alcaldes estuvo relacionada con el promedio per cápita de enmiendas transferidas del Fondo Nacional de Salud, de enero de 2021 a octubre de 2024. Se utilizó la regresión logística jerárquica al controlar los factores individuales y políticos de los candidatos, y los factores socioeconómicos y demográficos municipales. Los resultados indican que todos los modelos y recortes presentaron una asociación positiva y estadística significativa entre el aumento de los recursos de enmiendas dirigidas a la salud y la probabilidad de reelección de alcaldes en 2024. Se destaca que los valores de las enmiendas fueron más altos en municipios de hasta 20.000 habitantes en comparación con los otros municipios. Es posible que las enmiendas consoliden la imagen de gestores municipales como capaces de atraer recursos, lo cual favorece el apoyo de los electores en un contexto de contingencia de gastos federales. El uso político de las enmiendas contrasta con los criterios adoptados para la transferencia de recursos programáticos del SUS y puede debilitar la coordinación del Ministerio de Salud.

## Introduction

Since 2016, Brazil experiences a scenario of austerity and changes in budget regulation. There has been an increase in the share of parliamentary amendments (PAs) in Federal Government’s expenses ^(^ ^1^. PAs represent greater parliamentary protagonism in the allocation of federal resources and are analyzed in the literature under three main approaches: (i) governability instrument to pass the Executive branch’s agenda in the Brazilian National Congress ^(^ ^2^ ^,^ ^3^; (ii) strategy to favor the reelection of federal deputies ^(^ ^4^ ^,^ ^5^; and (iii) a tool to meet the political agenda of deputies and electoral demands, with an impact on public policies ^(^ ^6^.

Studies on the field of health show the implications of federal expenses for PAs, which became mandatory in 2015. Most PAs are transferred from the Brazilian Ministry of Health to the municipalities to fund the Brazilian Unified National Health System (SUS, acronym in Portuguese) ^(^ ^7^. The allocation of these resources increases regional inequity ^(^ ^8^, causes fragmentation and fluctuations in municipal revenues ^7^, and modifies the pattern of health expenses ^(^ ^9^. However, the relationship between PAs focused on the SUS and the reelection of mayors, whose political alignment favors the renewal of parliamentary term ^(^ ^10^, has been poorly explored.

Municipal administrators and deputies are concerned with health services since the delivery of public assets are easily identifiable by voters ^(^ ^11^. Mayors who raise more PAs for the SUS expand municipal resources, which can favor the fulfillment of specific local demands and contribute to an image of commitment to the population.

This article analyzes the association between the PAs destined to SUS and the reelection of mayors in the 2024 municipal elections, discussing meanings and implications for health policy in Brazil. This is based on the hypothesis that mayors who raise significant volumes of these PAs have competitive advantages for reelection.

## Method

### Type of study

This is an observational, exploratory, and descriptive study, conducted based on secondary sources of public access.

### Dependent variable

The dependent variable is the reelection of mayors who completed their term (2021-2024) and participated in the 2024 municipal elections. This variable is binary: 0 represents non-reelection, and 1 represents reelection.

### Independent variable

The independent variable is the per capita average of the amounts transferred through PAs from the Brazilian National Health Fund (NHF) to the municipalities, from January 2021 to October 2024. These amounts were adjusted for inflation, using the Broad Consumer Price Index (IPCA, acronym in Portuguese) of January 2024.

### Control variables

Based on the literature ^12,13^, the control variables were divided into two categories: (i) individual and political of candidates: gender of the mayor, political party (Center political parties, Workers’ Party - PT, or Liberal Party - PL, acronyms in Portuguese) and change of party affiliation between 2020 and 2024; (ii) socioeconomic and demographic aspects of the municipalities: status of state capital, location, proportion of families with income of up to half a minimum wage (2023), estimated population (2022), and gross domestic product (GDP) per capita adjusted for inflation (2019 amounts adjusted using the IPCA). Additional details are shown at Supplementary Material (Table S1 and Box S1; https://cadernos.ensp.fiocruz.br/static//arquivo/supl-e00220924_4558.pdf).

### Data source

Data on individual and political characteristics were obtained from the Superior Electoral Court (TSE, acronym in Portuguese) ^(^ ^14^, demographic and economic information came from the Brazilian Institute of Geography and Statistics (IBGE, acronym in Portuguese) ^(^ ^15^ and the Department of Appraisal and Management of *Cadastro Único* Information ^(^ ^16^. Data on PAs expenditures paid to municipalities were obtained from NHF ^(^ ^17^. Information was retrieved in October 2024.

### Spatial cutout

The analysis included 2,818 municipalities whose mayors elected in 2020 completed their term and ran for reelection in 2024. The demographic characteristics of these municipalities are detailed in the Supplementary Material (Figure S1 and Table S2; https://cadernos.ensp.fiocruz.br/static//arquivo/supl-e00220924_4558.pdf).

### Analysis model

A binomial logistic regression model was used to estimate the probability of reelection (0 or 1) ^(^ ^18^. The model included a two-level hierarchical structure, considering the municipal and state levels. This approach was chosen to capture the contextual effects of each state, given that local policy in Brazil has high socio-spatial heterogeneity ^(^ ^19^ and that the provision of health services is influenced by regional characteristics ^(^ ^20^.

### Analysis strategy

Three strategies were applied: (i) analysis of all municipalities; (ii) analysis in municipalities of up to 20,000 inhabitants; and (iii) analysis in municipalities with a population ranging 20,000 to 50,000 inhabitants. This division enables us to demonstrate the association between PAs and reelection in different electoral and demographic contexts, especially in smaller municipalities, which receive proportionally greater volume of resources ^8^ ^,^ ^9^. The models were estimated with the *glmmPQL* function of the *MASS* package in RStudio (https://rstudio.com/), incorporating fixed effects for the independent and control variables, as well as random effects for the states.

## Results

**Figure 1:**
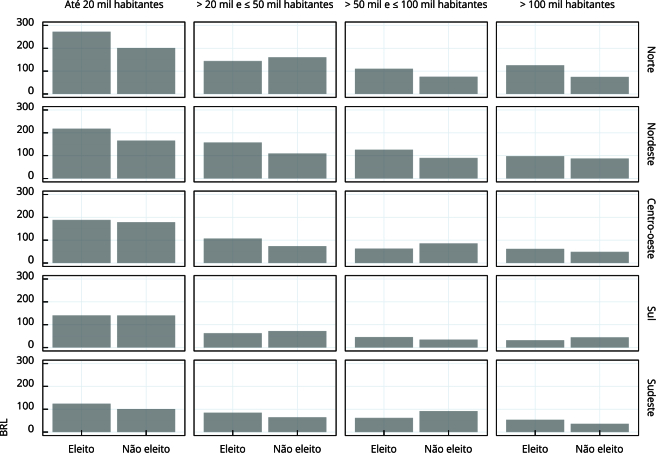
Average per capita amount (BRL) of parliamentary amendments aimed at health (2021-2024) by performance of candidates for municipal reelection in 2024, region of the country and population groups of municipalities.

Source: Brazilian Institute of Geography and Statistics ^15^ and Brazilian National Health Fund ^17^.

The distribution of the average amount of parliamentary amendments for health among the municipalities was significantly inequal (Figure 1). Disparities were observed between regions, population sizes, and electoral status of mayors. The town halls of the North and Northeast regions received, proportionally, the largest volumes, and the municipalities of up to 20,000 inhabitants were the most benefited.

It is also observed that mayors reelected in 2024 receive, on average, higher amounts of parliamentary amendments (Figure 1). Supplementary Material (Table S3; https://cadernos.ensp.fiocruz.br/static//arquivo/supl-e00220924_4558.pdf) shows the details of this distribution.

Table 1 shows the relation between these figures and the electoral success of the mayors, considering political, individual, socioeconomic, and demographic factors. Across all models, the per capita average of the amendments for health (2021-2024) is positively associated with reelection. In the general model, which includes all municipalities, the increase in the per capita amount was associated with the highest chance of electoral success. Models focused on municipalities with different population sizes corroborated this finding. In the Supplementary Material (https://cadernos.ensp.fiocruz.br/static//arquivo/supl-e00220924_4558.pdf), Table S4 and Figure S2 detail the reelection rates, while Table S5 the descriptive statistics.

**Table 1:** Factors associated with the reelection of mayors in 2024.

Predictive variables	All municipalities			Until 20,000 inhabitants			> 20,000 and ≤ 50,000 inhabitants		
	OR	95%CI	p-value	OR	95%CI	p-value	OR	95%CI	p-value
Intercept	0.80	0.20-3.26	0.762	1.04	0.10-11.33	0.973	0.00	0.00-4.34	0.100
Total per capita amendments for health 2021-2024	1.01	1.00-1.01	< 0.001	1.01	1.00-1.01	< 0.001	1.01	1.00-1.01	0.002
Females	0.71	0.54-0.94	0.018	0.62	0.44-0.88	0.008	0.71	0.39-1.29	0.270
Political party									
Center political parties	0.92	0.70-1.19	0.517	0.77	0.55-1.08	0.129	1.10	0.62-1.96	0.737
PL	1.58	0.99-2.52	0.056	1.12	0.63-1.99	0.705	3.74	1.02-13.73	0.050
PT	0.66	0.40-1.09	0.107	0.55	0.30-1.02	0.060	0.81	0.29-2.28	0.688
Change of party affiliation	0.98	0.80-1.21	0.882	1.01	0.78-1.31	0.932	0.69	0.43-1.12	0.135
GDP *per capita*	1.00	1.00-1.00	0.430	1.00	0.99-1.00	0.313	1.00	0.99-1.01	0.723
Families with half a minimum wage per capita	1.00	1.00-1.00	0.450	1.00	1.00-1.00	0.531	1.00	1.00-1.01	0.629
Population log	1.39	1.07-1.81	0.013	1.49	0.88-2.54	0.142	7.14	0.84-61.00	0.076
Status of state capital	0.41	0.10-1.64	0.210						
Location *									
South	0.52	0.29-0.92	0.026	0.35	0.18-0.69	0.004	0.93	0.33-2.65	0.894
Northeast	0.84	0.47-1.51	0.544	0.60	0.29-1.23	0.155	1.34	0.43-4.21	0.606
North	0.56	0.29-1.06	0.072	0.31	0.14-0.69	0.007	1.00	0.29-3.44	0.996
Southeast	0.70	0.41-1.21	0.194	0.55	0.29-1.07	0.077	0.95	0.37-2.40	0.904
Random effect									
σ^2^	3.29			3.29			3.29		
τ00	0.03_sigla_uf_			0.02_sigla_uf_			0.00_sigla_uf_		
ICC	0.01			0.01			0.00		
N	26_sigla_uf_			26_sigla_uf_			26_sigla_uf_		
Observations	2,818			1,967			534		
Marginal R^2^/Conditional R^2^	0.106/0.114			0.129/0.133			0.155/0.155		

95%CI: 95% confidence interval; GDP: gross domestic product; ICC: intraclass correlation coefficient; N: number of Brazilian states; OR: odds ratio; PL: Liberal Party; PT: Workers Party; sigla_uf: identification of the state to which the municipality belongs.

Source: TSE Open Data Portal ^14^; Brazilian Institute of Geography and Statistics ^15^; Department of Appraisal and Management of *Cadastro Único* Information ^16^; Brazilian National Health Fund ^17^.

Note: in bold are statistically significant p-values (p < 0.05).

* The Central-West region was used as the reference category of the model.

**Figure 2:**
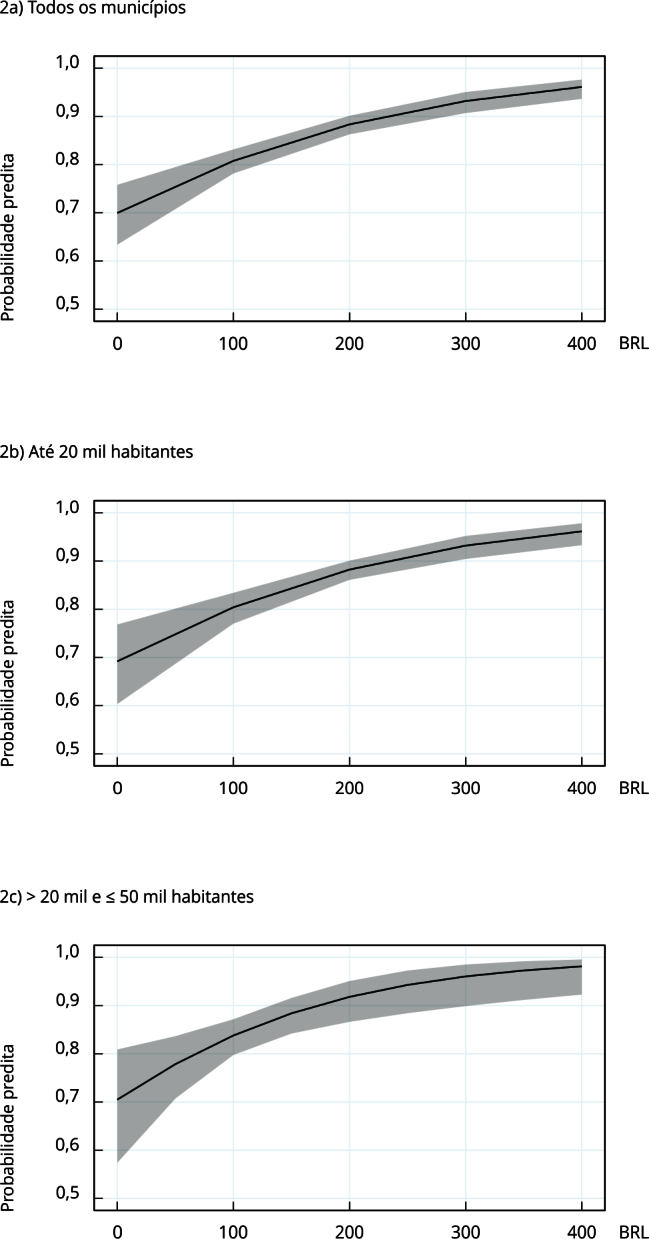
Relationship between average per capita amounts (BRL) of parliamentary amendments aimed at health (2021-2024) and the probability of reelection of mayors in 2024 by population groups of Brazilian municipalities.

Source: Brazilian Institute of Geography and Statistics ^15^; Department of Appraisal and Management of *Cadastro Único* Information ^16^; Brazilian National Health Fund ^17^.

Figure 2 shows graphs of the predicted probability of reelection as a function of the per capita amount of amendments for health, according to the estimated models. The curves indicate a progressive increase in the probability of reelection with the increase in the total figures of the PAs. In Figure 2a, which includes all municipalities, for each increase of 100 units in the per capita amount, the probability of reelection increases from approximately 0.7 to 0.8.

Robustness tests included models with the average of the amendments transformed into a categorical variable by quintiles, with consistent results (Supplementary Material - Figure S3; https://cadernos.ensp.fiocruz.br/static//arquivo/supl-e00220924_4558.pdf). Linear regression models, using the same variables as in Table 1, confirmed the positive association of amendments with reelection. Moreover, a model was developed with all municipalities with the independent normalized variable (log), in which the results remained consistent. Supplementary Material (Tables S6, S7 and S8; https://cadernos.ensp.fiocruz.br/static//arquivo/supl-e00220924_4558.pdf) shows the details.

## Discussion

This study suggests that PAs focusing on SUS played a relevant role in consolidating electoral support during the 2024 municipal elections. In Brazil, the rate of reelection of mayors reached an all-time high, peaking 83% success ^(^ ^12^. This increase may be linked to the strengthening of the Legislative branch after 2015, resulting in the expansion of resources transferred to municipalities via amendments ^(^ ^9^.

The literature indicates that financial resources influence political success, either by financing electoral campaigns ^(^ ^21^ or by supporting public policies ^(^ ^22^. Similarly, this research demonstrates that PAs focused on the SUS are related to greater success of mayor candidates, regardless of political party affiliation or individual characteristics. Possibly, PAs consolidate the image of the mayor as an “efficient administrator” or “capable of raising financial resources”, influencing the choice of voters. Thus, these amendments stand out as a tool for fundraising and political strategy in the local electoral context. The ability to articulate political support and guarantee these resources tends to positively influence the electoral result, especially in small municipalities.

Such ability to raise funds becomes even more relevant in a scenario of contingency of federal health expenses ^(^ ^23^. Given this context, along with the enforcement of PAs by the Federal Government, the SUS becomes increasingly dependent on political articulations to ensure its financing ^(^⁷^)^. Furthermore, the discretionary nature of PAs favors transfers to municipalities with a high concentration of voters or governed by mayors aligned with certain parliamentarians ^(^ ^24^. The disparities in the amounts of the amendments between municipalities can encourage disputes for resources, bringing electoral benefits to the managers of the most favored locations. This allocation mechanism contrasts with the criteria historically defined in an agreed manner between the Brazilian Ministry of Health and the other spheres of SUS management and with efforts at equitable distribution ^(^ ^8^. 

PAs also change the pattern of intergovernmental relations instituted in the process of implementing the SUS. In the Brazilian federative arrangement, with a predominance of small municipalities and regional inequalities, the organization of the provision of services depends on a collaborative and regionalized agreement between the different governmental spheres. The negotiation of regular and program-based funding enabled the Federal Government to coordinate priority policies and redistribute resources to poorer areas ^(^ ^20^. However, the competition for amendments splits the resources between municipalities, weakening the coordination capacity of the Brazilian Ministry of Health, since the fundings are mediated by relationships established between mayors and deputies, often on the margins of the federative instances of the SUS. Although PAs can create political dividends for its articulators, these gains do not necessarily translate into collective benefits and cannot guarantee the principles of universality and equity of health policy in Brazil.

This investigation has three main limitations. The time frame (2021-2024) can restrict the analysis to short-term and context-specific relationships. Furthermore, by considering only mayors who ran for reelection, selection bias can be introduced. Finally, the exclusive focus on amendments aimed at health disregards other sectors relevant to electoral performance.

The findings point out the need for further studies, employing different methodological approaches to better understand the causal mechanisms of the identified association. Among the aspects to be investigated, the degree of implementation of the amendments by the municipalities stands out, influenced by administrative, bureaucratic, and political factors. In addition, how can the allocation of these resources to health, in areas with greater or lesser visibility for the voter, have a different impact on the chances of reelection.
