# Supplementary material for: Parliamentary amendments aimed at the Brazilian Unified National Health System and the reelection of municipal mayors in Brazil in 2024
Source: Cad Saude Publica. 2025 May 19;41(4):e00220924. doi: 10.1590/0102-311XEN220924 (PMC12091854; doi:10.1590/0102-311XEN220924)
Supplement: Supplementary file 1 [file 1678-4464-csp-41-04-EN220924-s.pdf]

Quadro S1 Variáveis incorporadas na análise.

VARIÁVEL	TIPO DE VARIÁVEL	INFORMAÇÃO	FONTE
Reeleitos (2024)	Dependente	Indica se o prefeito candidato à reeleição nas eleições de 2024 conseguiu se reeleger.	TSE
mu Emendas totais para saúde per capita 2021 2024	Independente	A média dos valores pagos em emendas parlamentares ao Fundo Nacional da Saúde para os municípios, por habitantes entre 2021 e 2024.	Fundo Nacional da saúde
Sexo Feminino	Controle	Indica se o prefeito candiato nas eleições de 2024 é do sexo feminino.	TSE
Partido do Centrão	Controle	Indica se o prefeito candiato nas eleições de 2024 é de um partido político pertencente ao Centrão.	Da Silva (2022)
Partido Liberal (PL)	Controle	Indica se o prefeito candiato nas eleições de 2024 é do Partido Liberal (PL).	TSE
Partido dos Trabalhadores (PT)	Controle	Indica se o prefeito candiato nas eleições de 2024 é do Partido dos Trabalhadores (PT).	TSE
Troca de Partido	Controle	Indica se o prefeito candidato nas eleições de 2024 mudou de partido político em relação às eleições de 2020.	TSE
Capitais	Controle	Indica se o município é a capital de algum estado do país.	IBGE
Regiões do País	Controle	Indica a região do país em que o município está localizado.	IBGE
PIB per capita (2019)	Controle	Produto Interno Bruto per capita, a preços correntes (R$ 1,00) (2019), deflacionado baseado no IPCA de 2024.	IBGE
Famílias com meio salário mínimo per capita (2023)	Controle já	Quantidade de famílias com renda per capita mensal até meio salário-mínimo (Pobreza + Baixa renda) inscritas no Cadastro Único, por mil habitantes.	SAGICAD e IBGE
População (2022)	Controle	Indica a população do município em 2022, conforme os dados do Censo de 2022.	IBGE

Fonte: elaboração própria.

Tabela S1 Valores totais de emendas para a saúde (em bilhões de R$).

Ano	Valores totais de emendas para a saúde
2021	R$ 9.363.066.462
2022	R$ 9.231.710.018
2023	R$ 7.954.331.327
2024	R$ 10.773.543.173

Fonte: elaboração própria. Fundo Nacional da Saúde.

Nota: os valores de 2024 se referem aos valores pagos até outubro de 2024. Todos os valores foram deflacionados segundo o IPCA de janeiro de 2024.

Tabela S2 Total da população nos municípios analisados e a distribuição desses municípios por região e por faixa de tamanho populacional segundo o Censo do IBGE de 2022.

A) População total dos municípios incluídos na análise

Municípios analisados	População brasileira	% Municípios Analisados
Pop. 108.004.764	Pop. 203.080.756	53,18

B) Distribuição dos municípios analisados segundo as regiões

Região	Municípios Analisados	% Municípios Analisados
Nordeste	913	32,40
Sudeste	860	30,52
Sul	549	19,48
Norte	249	8,84
Centro-Oeste	247	8,77

C) Distribuição dos municípios analisados segundo o tamanho populacional

Porte Populacional	Municípios Analisados	% Municípios Analisados
Até 20 mil hab	1967	69,80
Superior a 20 mil até 50 mil hab	534	18,95
Superior a 50 mil até 100 mil hab	178	6,32
Acima de 100.000 hab	139	4,93

Fonte: elaboração própria. IBGE.

Tabela S3 Valores médios de emendas parlamentares para saúde per capita (2021-2024) segundo o desempenho dos candidatos à reeleição nas eleições municipais de 2024.

A) Valores médios de emendas parlamentares para saúde e desempenho dos candidatos à reeleição

**Situação**	**Valor médio de emenda parlamentar para saúde *per capita* (2021-2024)**
Eleito	155,62
Não Eleito	119,74

B) Valores médios de emendas parlamentares para saúde e desempenho dos candidatos à reeleição segundo as regiões do país

**Região**	**Situação**	**Valor médio de emenda parlamentar para saúde *per capita* (2021-2024)**
Centro-Oeste	Eleito	167,52
Centro-Oeste	Não Eleito	126,92
Nordeste	Eleito	195,15
Nordeste	Não Eleito	140,02
Norte	Eleito	211,66
Norte	Não Eleito	166,13
Sudeste	Eleito	109,22
Sudeste	Não Eleito	89,19
Sul	Eleito	121,89
Sul	Não Eleito	125,91

C) Valores médios de emendas parlamentares para saúde e desempenho dos candidatos à reeleição segundo porte populacional do município

**Tamanho Populacional**	**Situação**	**Valor médio de emenda parlamentar para sáude, per capita (2021-2024)**
Até 20 mil hab	Eleito	175,84
Até 20 mil hab	Não Eleito	137,94
Superior a 20 mil até 50 mil hab	Eleito	124,41
Superior a 20 mil até 50 mil hab	Não Eleito	90,93
Superior a 50 mil até 100 mil hab	Eleito	86,34
Superior a 50 mil até 100 mil hab	Não Eleito	83,55
Acima de 100.000 hab	Eleito	69,16
Acima de 100.000 hab	Não Eleito	54,87

D) Valores médios de emendas parlamentares para saúde

**Valor médio de emenda parlamentar para saúde *per capita* (2021-2024)**
149,56

E) Valores médios de emendas parlamentares para saúde segundo regiões do país

**Região**	**Valor médio de emenda parlamentar para saúde *per capita* (2021-2024)**
Centro-Oeste	162,43
Nordeste	188,81
Norte	204,35
Sudeste	105,38
Sul	122,88

F) Valores médios de emendas parlamentares para saúde segundo porte populacional do município

**Tamanho Populacional**	**Valor médio de emenda parlamentar para sáude, per capita (2021-2024)**
Até 20 mil hab	169,65
Superior a 20 mil até 50 mil hab	118,58
Superior a 50 mil até 100 mil hab	85,87
Acima de 100.000 hab	65,87

Fonte: elaboração própria. TSE, IBGE e Fundo Nacional de Saúde.

Tabela S4 Desempenho dos candidatos à reeleição nas eleições municipais de 2024.

A) Desempenho Geral

Situação	Quantidade por situação	Quantidade Total	Percentual por situação
Eleito	2342	2818	83,11
Não Eleito	476	2818	16,89

B) Desempenho segundo as regiões do país

Região	Situação	Quantidade por situação	Quantidade Total	Percentual por situação
Centro-Oeste	Eleito	216	247	87,45
Centro-Oeste	Não Eleito	31	247	12,55
Nordeste	Eleito	808	913	88,50
Nordeste	Não Eleito	105	913	11,50
Norte	Eleito	209	249	83,94
Norte	Não Eleito	40	249	16,06
Sudeste	Eleito	695	860	80,81
Sudeste	Não Eleito	165	860	19,19
Sul	Eleito	414	549	75,41
Sul	Não Eleito	135	549	24,59

C) Desempenho segundo porte populacional do município

Tamanho Populacional	Situação	Quantidade, por situação	Quantidade Total	Percentual, por situação
Até 20 mil hab	Eleito	1646	1967	83,68
Até 20 mil hab	Não Eleito	321	1967	16,32
Superior a 20 mil até 50 mil hab	Eleito	441	534	82,58
Superior a 20 mil até 50 mil hab	Não Eleito	93	534	17,42
Superior a 50 mil até 100 mil hab	Eleito	148	178	83,15
Superior a 50 mil até 100 mil hab	Não Eleito	30	178	16,85
Acima de 100.000 hab	Eleito	107	139	76,98
Acima de 100.000 hab	Não Eleito	32	139	23,02

Fonte: elaboração própria. IBGE e TSE.

Tabela S5 Estatísticas descritivas das variáveis empregadas no modelo.

**Variável**	**Tipo de Variáv el**	**Frequê ncia Não**	**Frequê ncia Sim**	**% Não**	**% Sim**	**Min.**	**1st Qu.**	**Media n**	**Mean**	**3rd Qu.**	**Max.**
Reeleitos	Depend ente	476	2342	16,89	83,11	-	-	-	-	-	-
mu Emendas totais para saúde per capita 2021 2024	Indepen dente	-	-	-	-	0	81,92	133,06	149,5 6	202,16	900,2 6
Sexo Feminino	Control e	2445	373	86,76	13,24	-	-	-	-	-	-
Partido do Centrão	Control e	937	1881	33,25	66,75	-	-	-	-	-	-
Partido Liberal (PL)	Control e	2603	215	92,37	7,63	-	-	-	-	-	-
Partido dos Trabalhadores (PT)	Control e	2679	139	95,07	4,93	-	-	-	-	-	-
Troca de Partido	Control e	1308	1510	46,42	53,58	-	-	-	-	-	-
Capitais	Control e	2803	15	99,47	0,53	-	-	-	-	-	-
Região Sul	Control e	2269	549	80,52	19,48	-	-	-	-	-	-
Região Nordeste	Control e	1905	913	67,6	32,4	-	-	-	-	-	-
Região Norte	Control e	2569	249	91,16	8,84	-	-	-	-	-	-
Região Sudeste	Control e	1958	860	69,48	30,52	-	-	-	-	-	-
PIB per capita	Control e	-	-	-	-	5,98	13,76	23,46	31,79	38,1	619,9 3
Famílias com meio salário mínimo per capita	Control e	-	-	-	-	10,9 1	129,7	201,6	206,0 8	275,77	549,7 1
População log	Control e	-	-	-	-	2,94	3,71	4,04	4,09	4,38	7,09

Fonte: elaboração própria. IBGE e TSE.

Nota: as estatísticas descritivas foram geradas a partir do banco de dados contendo todos os municípios analisados (2.818 municípios).

Tabela S6 Fatores associados à reeleição de prefeitos em 2024 (quintis de emendas parlamentares destinadas à saúde).

	Todos os municípios			Até 20 mil hab			Superior a 20 mil até 50 mil hab		
Variáveis Preditoras	*Odds Ratios*	*CI*	*p*	*Odds Ratios*	*CI*	*p*	*Odds Ratios*	*CI*	*p*
(Intercepto)	1,27	0,32 – 5,14	0,734	2,17	0,21 – 22,89	0,521	0,00	0,00 – 10,55	0,143
Emendas por quantis	1,31	1,18 – 1,45	**<0,001**	1,32	1,16 – 1,50	**<0,001**	1,36	1,08 – 1,72	**0,011**
Sexo Feminino	0,71	0,54 – 0,94	**0,016**	0,62	0,44 – 0,87	**0,006**	0,72	0,40 – 1,31	0,295
Partido do Centrão	0,92	0,70 – 1,19	0,517	0,77	0,55 – 1,07	0,125	1,13	0,63 – 1,99	0,690
PL	1,61	1,01 – 2,56	**0,046**	1,15	0,65 – 2,03	0,635	3,81	1,04 – 13,97	**0,047**
PT	0,67	0,41 – 1,10	0,112	0,56	0,30 – 1,02	0,061	0,78	0,27 – 2,20	0,638
Troca de Partido	0,98	0,79 – 1,20	0,815	0,99	0,77 – 1,29	0,966	0,70	0,43 – 1,13	0,151
PIB per capita	1,00	1,00 – 1,00	0,408	1,00	0,99 – 1,00	0,302	1,00	0,99 – 1,01	0,723
Famílias com meio salário mínimo per capita	1,00	1,00 – 1,00	0,421	1,00	1,00 – 1,00	0,547	1,00	1,00 – 1,01	0,579
População log	1,26	0,98 – 1,63	0,072	1,26	0,75 – 2,12	0,376	5,85	0,69 – 49,40	0,109
Capitais	0,51	0,13 – 1,97	0,328						
Região Sul	0,49	0,28 – 0,86	**0,015**	0,32	0,16 – 0,63	**0,002**	0,92	0,32 – 2,63	0,878
Região Nordeste	0,86	0,48 – 1,55	0,605	0,62	0,30 – 1,29	0,193	1,41	0,45 – 4,43	0,550
Região Norte	0,61	0,32 – 1,15	0,121	0,37	0,16 – 0,82	**0,017**	1,04	0,30 – 3,58	0,948
Região Sudeste	0,67	0,39 – 1,15	0,137	0,51	0,26 – 0,99	**0,048**	0,92	0,36 – 2,32	0,848
**Random Effects**									
σ^2	3,29			3,29			3,29		
τ_{00}	0,03 $_{sigla\_uf}$			0.02 $_{sigla\_uf}$			0.00 $_{sigla\_uf}$		
ICC	0,01			0,01			0.00		
N	26 $_{sigla\_uf}$			26 $_{sigla\_uf}$			26 $_{sigla\_uf}$		
Observações	2818			1967			534		
Marginal R^2 / Condicional R^2	0,080 / 0,089			0,102 / 0,108			0,126 / 0,126		

Fonte: elaboração própria.

Tabela S7 Fatores associados à reeleição de prefeitos em 2024 (modelos lineares) .

	**Todos os municípios**			**Até 20 mil hab**			**Superior a 20 mil até 50 mil hab**		
Variáveis Preditoras	*Estimates*	*CI*	*p*	*Estimates*	*CI*	*p*	*Estimates*	*CI*	*p*
(Intercepto)	0,65	0,46 – 0,84	**<0,001**	0,70	0,39 – 1,00	**<0,001**	-0,43	-1,76 – 0,89	0,520
mu Emendas totais para saúde per capita 2021 2024	0,00	0,00 – 0,00	**<0,001**	0,00	0,00 – 0,00	**<0,001**	0,00	0,00 – 0,00	**0,003**
Sexo Feminino	-0,05	-0,09 – -0,01	**0,014**	-0,07	-0,12 – -0,02	**0,006**	-0,05	-0,13 – 0,04	0,298
Partido do Centrão	-0,01	-0,05 – 0,02	0,455	-0,03	-0,08 – 0,01	0,103	0,01	-0,07 – 0,09	0,778
PL	0,06	0,00 – 0,12	**0,043**	0,02	-0,05 – 0,09	0,571	0,14	0,00 – 0,27	**0,046**
PT	-0,05	-0,12 – 0,02	0,157	-0,07	-0,15 – 0,01	0,089	-0,02	-0,18 – 0,13	0,766
Troca de Partido	-0,00	-0,03 – 0,03	0,966	0,00	-0,03 – 0,04	0,912	-0,05	-0,11 – 0,02	0,162
PIB per capita	-0,00	-0,00 – 0,00	0,331	-0,00	-0,00 – 0,00	0,234	0,00	-0,00 – 0,00	0,670
Famílias com meio salário mínimo per capita	0,00	-0,00 – 0,00	0,497	0,00	-0,00 – 0,00	0,677	0,00	-0,00 – 0,00	0,554
População log	0,04	-0,00 – 0,07	0,055	0,04	-0,03 – 0,11	0,268	0,25	-0,04 – 0,54	0,091
Capitais	-0,10	-0,30 – 0,10	0,314						
Região Sul	-0,10	-0,19 – -0,02	**0,021**	-0,15	-0,24 – -0,05	**0,004**	-0,01	-0,17 – 0,15	0,879
Região Nordeste	-0,02	-0,10 – 0,06	0,567	-0,05	-0,15 – 0,04	0,242	0,04	-0,13 – 0,21	0,618
Região Norte	-0,07	-0,16 – 0,02	0,122	-0,12	-0,23 – -0,02	**0,026**	0,01	-0,16 – 0,19	0,879
Região Sudeste	-0,06	-0,14 – 0,02	0,163	-0,08	-0,17 – 0,02	0,105	-0,02	-0,16 – 0,13	0,799
**Random Effects**									
σ^2	0,14			0,13			0,14		
τ_{00}	0,00 $_{sigla\_uf}$			0,00 $_{sigla\_uf}$			0.00 $_{sigla\_uf}$		
ICC	0,01			0.01					
N	26 $_{sigla\_uf}$			26 $_{sigla\_uf}$			26 $_{sigla\_uf}$		
Observações	2818			1967			534		
Marginal R^2 / Condicional R^2	0,040 / 0,048			0,050 / 0,059			0,053 /		

Fonte: elaboração própria.

Tabela S8 Fatores associados à reeleição de prefeitos em 2024 (emendas para a saúde normalizada).

	Todos os municípios		
Variáveis Preditoras	*Odds Ratios*	*CI*	*p*
(Intercepto)	0,11	0,01 – 0,89	**0,039**
mu Emendas saúde normalizado	1,79	1,44 – 2,22	**<0,001**
Sexo Feminino	0,70	0,53 – 0,93	**0,014**
Partido do Centrão	0,92	0,71 – 1,19	0,523
PL	1,59	1,00 – 2,53	0,053
PT	0,67	0,41 – 1,10	0,116
Troca de Partido	0,98	0,79 – 1,20	0,829
PIB per capita	1,00	1,00 – 1,00	0,591
Famílias com meio salário mínimo per capita	1,00	1,00 – 1,00	0,451
População log	1,40	1,06 – 1,85	**0,019**
Capitais	0,42	0,10 – 1,67	0,218
Região Sul	0,50	0,28 – 0,89	**0,021**
Região Nordeste	0,88	0,49 – 1,60	0,669
Região Norte	0,62	0,33 – 1,19	0,143
Região Sudeste	0,68	0,39 – 1,20	0,176
**Random Effects**			
σ^2	3,29		
$\tau_{00\ sigla\_uf}$	0,04		
ICC	0,01		
N $_{sigla\_uf}$	26		
O Observações	2816		
Marginal R^2 / Condicional R^2	0,078 / 0,088		

Fonte: elaboração própria.

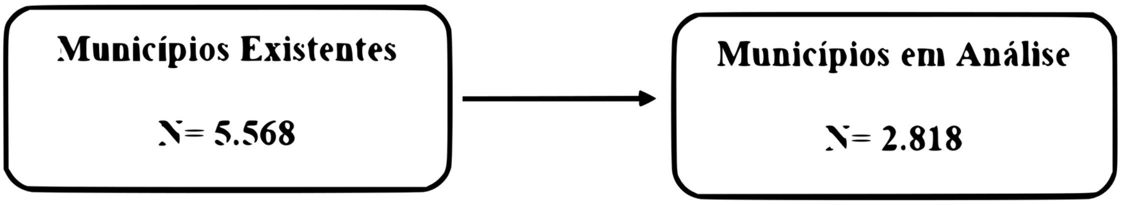

Figura S1 Municípios em que os prefeitos concorreram à reeleição em 2024.

Fonte: elaboração própria.

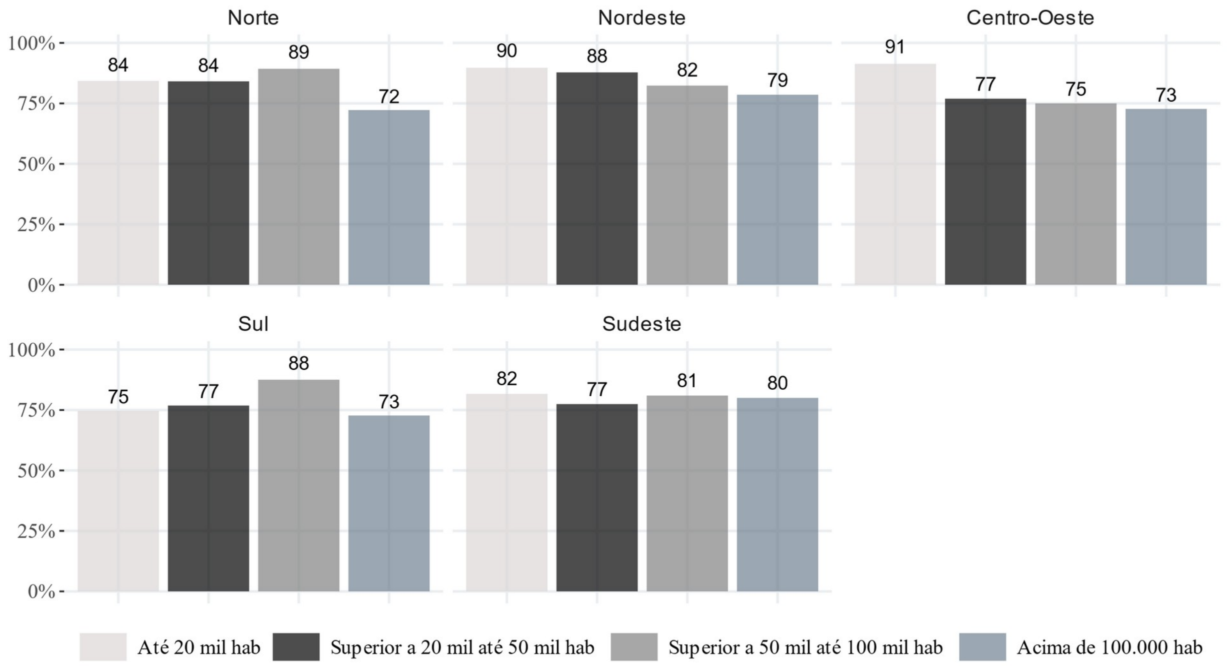

Figura S2 Percentual de prefeitos reeleitos segundo região e tamanho populacional do município.
Fonte: elaboração própria. IBGE e TSE.

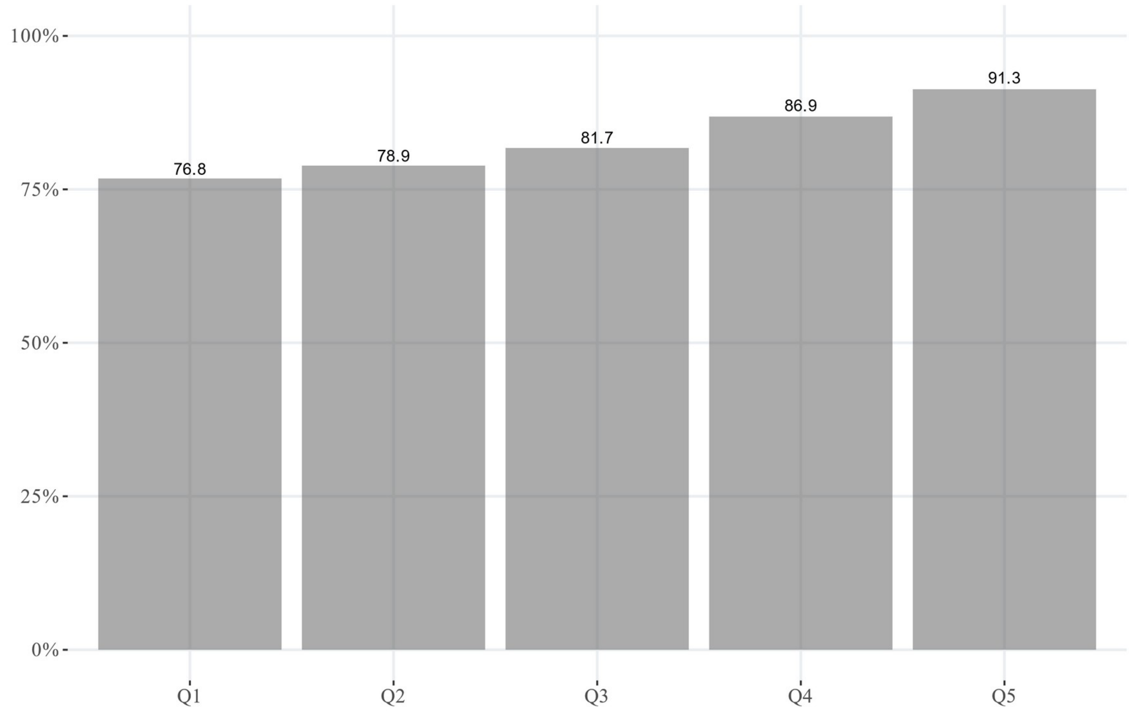

Figura S3 Percentual de prefeitos reeleitos segundo quintis de distribuição do valor médio de emenda parlamentar para saúde per capita (2021-2024).

Fonte: elaboração própria. IBGE e TSE.
