# Supplementary material for: Parliamentary amendments aimed at the Brazilian Unified National Health System and the reelection of municipal mayors in Brazil in 2024
Source: Cad Saude Publica. 2025 May 19;41(4):e00220924. doi: 10.1590/0102-311XEN220924 (PMC12091854; doi:10.1590/0102-311XEN220924)
Supplement: Translation

# Translation: Emendas parlamentares destinadas ao Sistema Único de Saúde e a reeleição de prefeitos municipais do Brasil em 2024

**DOI:** 10.1590/0102-311XPT220924

**Authors:** João Gabriel Ribeiro Pessanha Leal, Luciana Dias de Lima, Frederico Bertholini Santos Rodrigues, André Schimidt da Silva

**Affiliations:** 1 Escola Nacional de Saúde Pública Sergio Arouca, Fundação Oswaldo Cruz, Rio de Janeiro, Brasil.; 2 Instituto de Ciência Política, Universidade de Brasília, Brasília, Brasil.

**Keywords:** Financiamento dos Sistemas de Saúde, Sistema Único de Saúde, Poder Legislativo, Política, Municípios

## Abstract

No Brasil, a partir de 2016, observa-se aumento da participação das emendas parlamentares (EPs) no orçamento da União, transferidas do Ministério da Saúde aos municípios para o custeio do Sistema Único de Saúde (SUS). Este artigo analisa a associação entre as EPs destinadas ao SUS e a reeleição de prefeitos municipais em 2024. A pesquisa utilizou dados secundários públicos, envolvendo 2.818 municípios. A reeleição de prefeitos foi relacionada à média *per capita* de emendas transferidas do Fundo Nacional de Saúde, de janeiro de 2021 a outubro de 2024. Utilizou-se regressão logística hierárquica, controlando fatores individuais e políticos dos candidatos, e socioeconômicos e demográficos municipais. Os resultados indicam que em todos os modelos e recortes houve uma associação positiva e estatisticamente significativa entre o aumento dos recursos de emendas destinadas à saúde e a probabilidade de reeleição de prefeitos em 2024. Destaca-se que os valores de emendas foram consideravelmente maiores nos municípios de até 20 mil habitantes em comparação aos demais municípios. Possivelmente, as emendas consolidam a imagem dos gestores municipais como capazes de atrair recursos, fortalecendo seu apoio eleitoral em um contexto de contingenciamento de despesas federais. O uso político das emendas contrasta com os critérios adotados para o repasse de recursos programáticos do SUS e pode fragilizar a coordenação do Ministério da Saúde.

## Introdução

No Brasil, desde 2016, em um cenário de austeridade e mudanças na regulamentação orçamentária, observa-se o aumento da participação das emendas parlamentares (EPs) nas despesas da União ^(^ ^1^. As EPs expressam maior protagonismo parlamentar na alocação de recursos federais e são analisadas na literatura sob três principais enfoques: (i) instrumento de governabilidade para aprovar a pauta do Executivo no Congresso Nacional ^(^ ^2^ ^,^ ^3^; (ii) estratégia para favorecer a reeleição de deputados federais ^(^ ^4^ ^,^ ^5^; e (iii) ferramenta para atender à agenda política dos parlamentares e demandas eleitorais, com impacto nas políticas públicas ^(^ ^6^.

Na saúde, estudos evidenciam as implicações das despesas federais por EPs, que se tornaram de execução obrigatória em 2015. Em sua maioria, as emendas são transferidas do Ministério da Saúde aos municípios para custear o Sistema Único de Saúde (SUS) ^(^ ^7^. A alocação desses recursos aumenta a iniquidade regional ^(^ ^8^, causa fragmentação e flutuações das receitas municipais ^7^ e modifica o padrão de despesas em saúde ^(^ ^9^. Contudo, a relação entre EPs voltadas para o SUS e a reeleição de prefeitos, cujo alinhamento político favorece a renovação de mandatos parlamentares ^(^ ^10^, tem sido pouco explorada.

A saúde atrai gestores municipais e parlamentares pela entrega de bens públicos facilmente identificáveis pelos eleitores ^(^ ^11^. Prefeitos que captam mais EPs para o SUS expandem recursos municipais, o que pode favorecer o atendimento de demandas locais específicas e contribuir para uma imagem de comprometimento com a população.

Este artigo analisa a associação entre as EPs destinadas ao SUS e a reeleição de prefeitos nas eleições municipais de 2024, discutindo significados e implicações para a política de saúde no Brasil. Parte-se da hipótese de que prefeitos que captam volumes expressivos dessas EPs têm vantagens competitivas para a reeleição.

## Método

### Tipo de estudo

O estudo é observacional, de caráter exploratório e descritivo, realizado a partir de fontes secundárias de acesso público.

### Variável dependente

A variável dependente é a reeleição dos prefeitos que completaram o mandato (2021-2024) e participaram das eleições municipais de 2024. Essa variável é binária: 0 representa não reeleição, e 1 representa reeleição.

### Variável independente

A variável independente é a média *per capita* dos valores transferidos por EPs do Fundo Nacional de Saúde (FNS) aos municípios, de janeiro de 2021 a outubro de 2024. Esses valores foram ajustados pela inflação, utilizando o Índice Nacional de Preços ao Consumidor Amplo (IPCA) de janeiro de 2024.

### Variáveis de controle

As variáveis de controle, baseadas na literatura ^(^ ^12^ ^,^ ^13^, foram divididas em duas categorias: (i) individuais e políticas dos candidatos: sexo do prefeito, partido político (“Centrão”, Partido dos Trabalhadores - PT ou Partido Liberal - PL) e troca de partido entre 2020 e 2024; (ii) socioeconômicas e demográficas dos municípios: *status* de capital estadual, localização regional, proporção de famílias com renda de até meio salário mínimo (2023), população estimada (2022) e produto interno bruto (PIB) *per capita* ajustado pela inflação (valores de 2019 corrigidos pelo IPCA). Detalhes adicionais encontram-se no Material Suplementar (Tabela S1 e Quadro S1; https://cadernos.ensp.fiocruz.br/static//arquivo/supl-e00220924_4558.pdf).

### Fonte de dados

Os dados sobre as características individuais e políticas foram obtidos do Tribunal Superior Eleitoral (TSE) ^(^ ^14^, e as informações demográficas e econômicas vieram do Instituto Brasileiro de Geografia e Estatística (IBGE) ^(^ ^15^ e da Secretaria de Avaliação e Gestão da Informação do Cadastro Único ^(^ ^16^. Os dados sobre as despesas com EP pagas aos municípios foram obtidos do FNS ^(^ ^17^. A coleta foi realizada em outubro de 2024.

### Recorte espacial

A análise incluiu 2.818 municípios cujos prefeitos eleitos em 2020 concluíram o mandato e concorreram à reeleição em 2024. As características demográficas desses municípios estão detalhadas no Material Suplementar (Figura S1 e Tabela S2; https://cadernos.ensp.fiocruz.br/static//arquivo/supl-e00220924_4558.pdf).

### Modelo de análise

Foi utilizado um modelo de regressão logística binomial para estimar a probabilidade de reeleição (0 ou 1) ^(^ ^18^. O modelo incluiu uma estrutura hierárquica de dois níveis, considerando o nível municipal e o estadual. Essa abordagem foi escolhida para capturar os efeitos contextuais de cada estado, dado que a política local no Brasil apresenta alta heterogeneidade socioespacial ^(^ ^19^ e que a provisão de serviços de saúde é influenciada por características regionais ^(^ ^20^.

### Estratégia de análise

Foram aplicadas três estratégias: (i) análise de todos os municípios; (ii) análise de municípios com até 20 mil habitantes; e (iii) análise de municípios com população superior a 20 mil até 50 mil habitantes. Esta divisão permite demonstrar a associação entre EPs e reeleição em diferentes contextos eleitorais e demográficos, especialmente em municípios de pequeno porte populacional, que recebem proporcionalmente maior volume desses recursos ^8^ ^,^ ^9^. Os modelos foram estimados com a função *glmmPQL* do pacote *MASS* no RStudio (https://rstudio.com/), incorporando efeitos fixos para as variáveis independentes e de controle, além de efeitos aleatórios para os estados.

## Resultados

A distribuição do valor médio das emendas parlamentares para a saúde entre os municípios mostrou significativa desigualdade (Figura 1). Disparidades foram observadas entre regiões, tamanhos populacionais e situação eleitoral dos prefeitos. As prefeituras das regiões Norte e Nordeste receberam, proporcionalmente, os maiores volumes, e os municípios de até 20 mil habitantes foram os mais beneficiados.

**Figura 1:** Valor médio *per capita* (BRL) das emendas parlamentares destinadas à saúde (2021-2024) por desempenho dos candidatos à reeleição municipal em 2024, região do país e faixas populacionais dos municípios.

Fonte: Instituto Brasileiro de Geografia e Estatística ^15^ e Fundo Nacional de Saúde ^17^.

Observa-se, ainda, a predominância de prefeitos reeleitos que receberam, em média, valores mais altos de emendas parlamentares (Figura 1). Detalhes dessa distribuição estão no Material Suplementar (Tabela S3; https://cadernos.ensp.fiocruz.br/static//arquivo/supl-e00220924_4558.pdf).

A Tabela 1 apresenta a relação entre esses valores e o sucesso eleitoral dos prefeitos, considerando fatores políticos, individuais, socioeconômicos e demográficos. Em todos os modelos, a média *per capita* das emendas para saúde (2021-2024) está positivamente associada com a probabilidade de reeleição. No modelo geral, que inclui todos os municípios, o aumento no montante *per capita* esteve associado à maior chance de sucesso eleitoral. Modelos focados em municípios com tamanhos populacionais distintos corroboraram esse achado. No Material Suplementar (https://cadernos.ensp.fiocruz.br/static//arquivo/supl-e00220924_4558.pdf), a Tabela S4 e a Figura S2 detalham as taxas de reeleição, enquanto as estatísticas descritivas estão na Tabela S5.

**Tabela 1:** Fatores associados à reeleição de prefeitos em 2024.

Variáveis preditoras	Todos os municípios			Até 20 mil habitantes			> 20 mil e ≤ 50 mil habitantes		
	OR	IC95%	Valor de p	OR	IC95%	Valor de p	OR	IC95%	Valor de p
Intercepto	0,80	0,20-3,26	0,762	1,04	0,10-11,33	0,973	0,00	0,00-4,34	0,100
Emendas totais para saúde *per capita* 2021-2024	1,01	1,00-1,01	< 0,001	1,01	1,00-1,01	< 0,001	1,01	1,00-1,01	0,002
Sexo feminino	0,71	0,54-0,94	0,018	0,62	0,44-0,88	0,008	0,71	0,39-1,29	0,270
Partido político									
“Centrão”	0,92	0,70-1,19	0,517	0,77	0,55-1,08	0,129	1,10	0,62-1,96	0,737
PL	1,58	0,99-2,52	0,056	1,12	0,63-1,99	0,705	3,74	1,02-13,73	0,050
PT	0,66	0,40-1,09	0,107	0,55	0,30-1,02	0,060	0,81	0,29-2,28	0,688
Troca de partido	0,98	0,80-1,21	0,882	1,01	0,78-1,31	0,932	0,69	0,43-1,12	0,135
PIB *per capita*	1,00	1,00-1,00	0,430	1,00	0,99-1,00	0,313	1,00	0,99-1,01	0,723
Famílias com meio salário mínimo *per capita*	1,00	1,00-1,00	0,450	1,00	1,00-1,00	0,531	1,00	1,00-1,01	0,629
População *log*	1,39	1,07-1,81	0,013	1,49	0,88-2,54	0,142	7,14	0,84-61,00	0,076
*Status* de capital estadual	0,41	0,10-1,64	0,210						
Localização regional *									
Sul	0,52	0,29-0,92	0,026	0,35	0,18-0,69	0,004	0,93	0,33-2,65	0,894
Nordeste	0,84	0,47-1,51	0,544	0,60	0,29-1,23	0,155	1,34	0,43-4,21	0,606
Norte	0,56	0,29-1,06	0,072	0,31	0,14-0,69	0,007	1,00	0,29-3,44	0,996
Sudeste	0,70	0,41-1,21	0,194	0,55	0,29-1,07	0,077	0,95	0,37-2,40	0,904
**Efeito *random***									
σ^2^	3,29			3,29			3,29		
τ00	0,03_sigla_uf_			0,02_sigla_uf_			0,00_sigla_uf_		
ICC	0,01			0,01			0,00		
N	26_sigla_uf_			26_sigla_uf_			26_sigla_uf_		
Observações	2.818			1.967			534		
Marginal R^2^/Condicional R^2^	0,106/0,114			0,129/0,133			0,155/0,155		

IC95%: intervalo de 95% de confiança; ICC: coeficiente de correlação intraclasse; N: número de estados brasileiros; OR: *odds ratio*; PIB: produto interno bruto; PL: Partido Liberal; PT: Partido dos Trabalhadores; sigla_uf: identificação do estado ao qual o município pertence.

Fonte: Portal de Dados Abertos do TSE ^14^; Instituto Brasileiro de Geografia e Estatística ^15^; Secretaria de Avaliação, Gestão da Informação e Cadastro Único ^16^; Fundo Nacional de Saúde ^17^.

Nota: em negrito estão valores de p estatisticamente significativos (p < 0,05).

* A Região Centro-oeste foi utilizada como categoria de referência do modelo.

A Figura 2 apresenta gráficos de probabilidade predita de reeleição em função do montante *per capita* de emendas para a saúde, conforme os modelos estimados. As curvas indicam aumento progressivo na probabilidade de reeleição com o incremento nos valores das emendas. Na Figura 2a, que inclui todos os municípios, observa-se que, para cada aumento de 100 unidades no valor *per* capita, a probabilidade de reeleição cresce de aproximadamente 0,7 para 0,8.

**Figura 2:** Relação entre os valores médios *per capita* (BRL) das emendas parlamentares destinadas à saúde (2021-2024) e a probabilidade de reeleição de prefeitos em 2024 por faixas populacionais dos municípios do Brasil.

Fonte: Portal de Dados Abertos do TSE ^14^; Instituto Brasileiro de Geografia e Estatística ^15^; Secretaria de Avaliação, Gestão da Informação e Cadastro Único ^16^; Fundo Nacional de Saúde ^17^.

Testes de robustez incluíram modelos com a média das emendas transformada em variável categórica por quintis, com resultados consistentes (Material Suplementar - Figura S3; https://cadernos.ensp.fiocruz.br/static//arquivo/supl-e00220924_4558.pdf). Modelos de regressão linear, utilizando as mesmas variáveis da Tabela 1, confirmaram a associação positiva das emendas com a reeleição. Além disso, foi elaborado um modelo com todos os municípios com a variável independente normalizada (*log*), nos quais os resultados permaneceram consistentes. Os detalhes podem ser vistos no Material Suplementar (Tabelas S6, S7 e S8; https://cadernos.ensp.fiocruz.br/static//arquivo/supl-e00220924_4558.pdf).

## Discussão

Este estudo sugere que as EPs destinadas ao SUS desempenharam um papel relevante na consolidação de apoio eleitoral durante as eleições municipais de 2024. No Brasil, o índice de reeleição de prefeitos atingiu um recorde histórico, alcançando 83% de sucesso ^(^ ^12^. Esse aumento pode estar vinculado ao fortalecimento do Legislativo após 2015, que resultou na ampliação dos recursos transferidos aos municípios por meio de emendas ^(^ ^9^.

A literatura indica que os recursos financeiros influenciam o desempenho político, seja pelo financiamento de campanhas eleitorais ^(^ ^21^, seja pelo suporte a políticas públicas ^(^ ^22^. De forma semelhante, esta pesquisa demonstra que as EPs voltadas para o SUS estão relacionadas a um maior êxito de prefeitos candidatos, independentemente do partido político ou de suas características individuais. Possivelmente, as emendas consolidam a imagem do prefeito como “gestor eficiente” ou “capaz de atrair recursos”, influenciando a escolha dos eleitores. Assim, essas emendas destacam-se como ferramenta para captação de recursos e estratégia política no contexto eleitoral local. A habilidade de articular apoio político e garantir esses recursos tende a influenciar positivamente no resultado eleitoral, especialmente nos pequenos municípios.

Essa capacidade de captação de recursos ganha ainda mais destaque em um cenário de contingenciamento das despesas federais em saúde ^(^ ^23^. Nesse contexto, aliado à execução impositiva de EPs pelo Governo Federal, o SUS torna-se cada vez mais dependente de articulações políticas para assegurar seu financiamento ^(^ ^7^. Outrossim, o caráter discricionário das emendas favorece repasses a municípios com alta concentração de eleitores ou governados por prefeitos alinhados a determinados parlamentares ^(^ ^24^. As disparidades nos valores das emendas entre municípios podem incentivar disputas por recursos, trazendo benefícios eleitorais aos gestores das localidades mais favorecidas. Esse mecanismo de alocação contrasta com os critérios historicamente definidos de forma pactuada entre o Ministério da Saúde e as demais esferas de gestão do SUS e com esforços de distribuição equitativa ^(^ ^8^. 

As emendas também alteram o padrão de relações intergovernamentais instituído no processo de implantação do SUS. No arranjo federativo brasileiro, com predominância de pequenos municípios e desigualdades regionais, a organização da oferta de serviços depende de um acordo colaborativo e regionalizado entre os diferentes níveis de gestão. A negociação de repasses regulares e programáticos permitiu ao Governo Federal coordenar políticas prioritárias e redistribuir recursos para áreas mais carentes ^(^ ^20^. Contudo, a competição por emendas fragmenta a alocação de recursos entre municípios, enfraquecendo a capacidade de coordenação do Ministério da Saúde, já que os repasses são mediados por relações estabelecidas entre prefeitos e parlamentares, muitas vezes à margem das instâncias de negociação federativas do SUS. Embora as EPs possam gerar dividendos políticos para seus articuladores, esses ganhos não necessariamente se traduzem em benefícios coletivos, sendo insuficientes para garantir os princípios de universalidade e equidade da política de saúde no Brasil.

Este trabalho apresenta três limitações principais. O recorte temporal (2021-2024) pode restringir a análise das relações de curto prazo e contextos específicos. Ademais, ao considerar apenas prefeitos que disputaram a reeleição, pode-se introduzir viés de seleção. Por fim, o foco exclusivo em emendas destinadas à saúde desconsidera outros setores relevantes para o desempenho eleitoral.

Os achados indicam a necessidade de novos estudos, com diferentes abordagens metodológicas, para melhor compreensão dos mecanismos causais da associação identificada. Dentre os aspectos a serem investigados, destaca-se o grau de implementação das emendas pelos municípios, influenciado por fatores administrativos, burocráticos e políticos. Além disso, a destinação desses recursos para a saúde, em áreas com maior ou menor visibilidade para o eleitor, pode impactar de forma diferenciada as chances de reeleição.
